# arterioscope.sim: Enabling Simulations of Blood Flow and Its Impact on Bioimpedance Signals

**DOI:** 10.3390/bioengineering11121273

**Published:** 2024-12-15

**Authors:** Thomas Krispel, Vahid Badeli, Alireza Jafarinia, Alice Reinbacher-Köstinger, Christian Tronstad, Sascha Ranftl, Ørjan Grottem Martinsen, Håvard Kalvoy, Jonny Hisdal, Manfred Kaltenbacher, Thomas Hochrainer

**Affiliations:** 1 Institute for Fundamentals and Theory in Electrical Engineering, Graz University of Technology, 8010 Graz, Austria; thomas.krispel@tugraz.at (T.K.); alice.koestinger@tugraz.at (A.R.-K.); manfred.kaltenbacher@tugraz.at (M.K.); 2 Institute of Strength of Materials, Graz University of Technology, 8010 Graz, Austria; alireza.jafarinia@tugraz.at (A.J.); hochrainer@tugraz.at (T.H.); 3 Department of Clinical and Biomedical Engineering, Oslo University Hospital, 0372 Oslo, Norway; chrton@ous-hf.no (C.T.); o.g.martinsen@fys.uio.no (Ø.G.M.); havard.kalvoy@ous-hf.no (H.K.); 4 Institute of Theoretical and Computational Physics, Graz University of Technology, 8010 Graz, Austria; ranftl@tugraz.at; 5 Department of Physics, University of Oslo, 0371 Oslo, Norway; 6 Department of Vascular Diseases at Oslo University Hospital, 0586 Oslo, Norway; jonny.hisdal@medisin.uio.no

**Keywords:** bioimpedance, blood flow, cardiovascular disease, impedance plethysmography, numerical simulation

## Abstract

Objectives: Early detection of cardiovascular diseases and their pre-existing conditions, arteriosclerosis and atherosclerosis, is crucial to increasing a patient’s chance of survival. While imaging technologies and invasive procedures provide a reliable diagnosis, they carry high costs and risks for patients. This study aims to explore impedance plethysmography (IPG) as a non-invasive and affordable alternative for diagnosis. Methods: To address the current lack of large-scale, high-quality impedance data, we introduce arterioscope.sim, a simulation platform that models arterial blood flow and computes the electrical conductivity of blood. The platform simulates bioimpedance measurements on specific body segments using patient-specific parameters. The study investigates how introducing arterial diseases into the simulation affects the bioimpedance signals. Results: The simulation results demonstrate that introducing atherosclerosis and arteriosclerosis leads to significant changes in the computed signals compared to simulations of healthy arteries. Furthermore, simulation of a patient-specific healthy artery strongly correlates with measured signals from a healthy volunteer. Conclusions and significance: arterioscope.sim effectively simulates bioimpedance signals in healthy and diseased arteries and highlights the potential of using these signals for early diagnosis of arterial diseases, offering a non-invasive and cost-effective alternative to traditional diagnostic methods.

## 1. Introduction

Cardiovascular diseases (CVDs) are the leading cause of death globally, with an especially high prevalence in western countries [1]. Early diagnosis is crucial for increasing a patient’s chances of survival. Diagnosis of major pre-existing conditions, such as arteriosclerosis and atherosclerosis, can allow for treatment before these conditions progress to more severe forms of CVD. Arteriosclerosis and atherosclerosis, denoting arterial wall stiffening and plaque formation at the wall, respectively, affect blood flow in the concerned body regions [2]. Detecting such changes in the arterial blood flow could enable early diagnosis of such conditions [3]. Current gold-standard diagnostic methods, including imaging and invasive procedures, have been instrumental in medical practice. However, these techniques incur substantial costs and present potential risks to patients. Those risks include infections [4], radiation exposure [5], and the formation of pseudoaneurysms [6]. Additionally, they are predominantly employed only after symptoms appear, thus not facilitating early-stage detection. There is a growing need for a quick, safe, non-invasive, and affordable method to complement current techniques.

**Figure 1:**
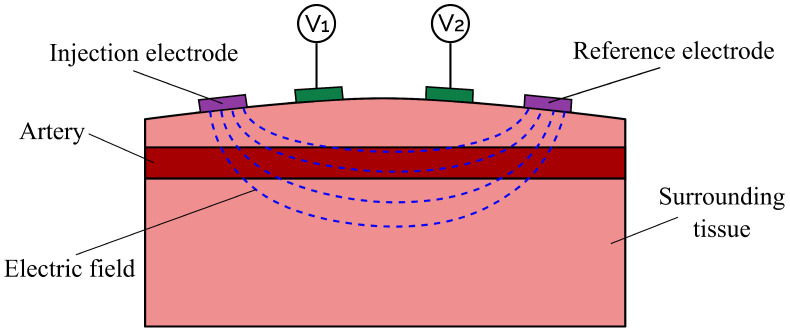
A setup for IPG measurements. An alternating current is injected into a body segment of interest via the injection electrode and reference electrode (**purple**), causing the formation of an electric field (**blue dashed line**). This leads to a difference in electric potential at the two pickup electrodes (**green**).

Bioimpedance signals offer a promising solution with these benefits. During bioimpedance measurements, such as impedance plethysmography (IPG) and impedance cardiography (ICG), a low-amplitude alternating current is injected into the body and the potential difference between pickup electrodes is measured to evaluate the transfer impedance [7]. The setup for an IPG measurement is shown in Figure 1. These non-invasive procedures are not dependent on high-cost technology, providing accessibility and the possibility of widespread application [8].

Bioimpedance changes during the cardiac cycle are caused by two main factors: arterial volume expansion and the flow-dependent electrical conductivity of blood. Blood has a higher electrical conductivity than bone and muscle [9]. Hence, the conductance of a body segment changes upon expansion of the arterial blood volume [10]. During the cardiac cycle, a pressure pulse wave is propagated through the vascular system causing local expansions of the arteries. These expansions are dependent on the vessel wall stiffness and vascular tone. Hence, changes in the vascular tone in combination with increased stiffness of the arterial wall are expected to leave a trace on bioimpedance signals [7]. Additionally, red blood cell (RBC) orientation is dependent on the current flow state, which causes anisotropy, inhomogeneity, and time-dependency of the electrical conductivity of blood [11]. These conductivity fluctuations are the second main contributor to bioimpedance changes during the cardiac cycle. Due to the flow-dependency of the electrical conductivity, changes in blood flow due to CVDs can also affect bioimpedance signals. However, the complex relationships between multiple physiological events and bioimpedance changes make the interpretation of these signals challenging. Therefore, large-scale studies of these dependencies are necessary to enable reliable diagnoses from the signals.

A promising prospect involves classification algorithms to detect CVDs or pre-existing conditions from the measured IPG signals [12]. However, training machine learning classification algorithms as well as conducting in-depth studies of the correlations between arterial defects and bioimpedance signals require a large number of high-quality measurements alongside verified diagnoses of potential CVDs. Acquiring such a substantial amount of data in clinical trials is costly and time-consuming. Numerical simulations can help overcome these difficulties by generating bioimpedance signals for virtual patients to support conventional data acquisition. Blood flow simulations in arteries enable the computation of the blood electrical conductivity, according to the novel model introduced in [11]. The computed electrical conductivity is needed in electrical simulations of the bioimpedance measurements to generate the according signals.

In this study, we introduce arterioscope.sim, a simulation platform consisting of a Computational Fluid Dynamics (CFD) module, in which arterial blood flow is simulated and the electrical conductivity of blood is computed, and a subsequent Computational Electroquasistatics (CEQS) module, in which the bioimpedance measurements are simulated. Its current focus is the simulation of blood flow in peripheral arteries and the corresponding IPG measurements, specifically of the popliteal artery (PA). The platform is parameterized, allowing the simulation of bioimpedance measurements on real and virtual patients. Additionally, arteriosclerosis and atherosclerosis at different progression levels are introduced into the model. Arteriosclerosis describes increases in arterial wall stiffness. Atherosclerosis is defined by plaque accumulations within the arterial flow, which alter the blood flow. In [13], similar CFD simulations of blood flow through deformed blood vessels were performed, resulting in a good match between simulation results and measurements. Eventually, the platform is able to generate IPG signals for healthy individuals as well as patients with CVDs. The main goal is to investigate if the generated impedance signals exhibit differences based on the health status of the artery. Such differences would be an indicator that the observed conditions can be diagnosed from bioimpedance measurements and that the simulation platform can assist in the necessary data acquisition.

By incorporating the most detailed model for the computation of the electrical conductivity of blood, arterioscope.sim also offers a more detailed simulation of bioimpedance measurement than previous models. Hence, more correlations between physiological events and bioimpedance signals can be concluded from the simulations. Through parametrization, the simulation pipeline can produce a large number of simulated signals efficiently. arterioscope.sim is not meant for direct use in clinical applications; rather, its purpose is to enable in-depth investigations into the applicability of bioimpedance measurements for monitoring and diagnosing CVD.

## 2. Materials and Methods

### 2.1. Governing Equations

#### 2.1.1. Blood Flow

Human blood is modeled as an incompressible fluid with constant density ρ. The arterial blood flow is described by the continuity equation

(1) ∇·u=0

and the momentum equation

(2) $$\rho \left[\frac{\partial u}{\partial t}+\left(u\;\cdot \;\nabla \right)u\right]=-\nabla p+\nabla \;\cdot \;\tau .$$

where u denotes the velocity vector, *p* denotes the pressure, and τ denotes the extra stress tensor [14]. While blood is often modeled as a Newtonian fluid, recent studies have shown that this simplification can lead to inaccuracies in both the flow field [15] and subsequently in the electrical conductivity [16]. We, therefore, treat blood as a non-Newtonian fluid, specifically as a generalized Newtonian fluid according to Carreau’s model. Hence, the isotropic dynamic viscosity η is defined as a function of the shear rate $\dot{\gamma }$, i.e.,

(3) $$\eta \left(\dot{\gamma }\right)=\eta_{\inf}+\left(\eta_{0}-\eta_{\inf}\right){\left[1+{\left(\lambda \dot{\gamma }\right)}^{2}\right]}^{(n-1)/2},$$

with the parameters $\eta_{\inf}$, $\eta_{0}$, λ, and *n*. In (3), $\eta_{\inf}$ and $\eta_{0}$ are the limits of the dynamic viscosity when the shear rate tends to infinity and zero, respectively. Furthermore, λ and *n* describe the transition between those limits. Those parameters were defined as functions of the hematocrit *H*, i.e., the volume fraction of RBCs in the suspension in [17,18]. Additionally, as introduced in [17,18], a yield stress is incorporated into the modeling, meaning that the stress experienced by blood must exceed the yield stress value $\tau_{y}$ for flow to occur. For such a generalized Newtonian fluid with yield stress, the extra stress tensor is defined as

(4) $$\begin{array}{cc} \tau =\tau_{y}\frac{1}{\dot{\gamma }}D+2\eta \left(\dot{\gamma }\right)D\;\; & \mathrm{if}\;\;|\tau |\geq \tau_{y} \end{array}$$

(5) $$\begin{array}{cc} D=0\;\; & \mathrm{if}\;\;|\tau |<\tau_{y}. \end{array}$$

The rate of deformation tensor D is defined as the symmetric part of the velocity gradient, i.e.,

(6) $$D=\frac{1}{2}\left(\nabla u+{\left(\nabla u\right)}^{T}\right).$$

The shear rate and the magnitude of the extra stress tensor are calculated by

(7) $$\dot{\gamma }=\sqrt{2\mathrm{tr}(D^{2})},$$

and

(8) $$\left|\tau \right|=\sqrt{\frac{\mathrm{tr}(\tau^{2})}{2}}.$$

#### 2.1.2. Electric Field and Potential

The electric field E is characterized by Gauss’s law

(9) $$\nabla \;\cdot \;E=\frac{\rho_{c}}{\varepsilon_{0}},$$

and Faraday’s law

(10) $$\nabla \times E=-\frac{\partial B}{\partial t}.$$

Here, $\rho_{c}$ denotes the charge density and $\varepsilon_{0}$ denotes the vacuum permittivity [19]. Due to the frequency of the cardiac cycle being significantly smaller than the current frequency, we may assume

(11) $$\nabla \times E=\frac{\partial B}{\partial t}\approx 0.$$

Hence, the electric field can be expressed in terms of the gradient of the electric potential *V* only, i.e.,

(12) E=−∇V.

Additionally, with a sinusoidal electric current, the conservation of electric charge is constituted in the frequency domain as

(13) $$\nabla \;\cdot \;J+j\omega \nabla \;\cdot \;D_{d}=0,$$

where J is the electric current density, ω is the angular frequency, $D_{d}$ denotes the electric displacement field, and j is the imaginary unit [20]. Using (12) and (13), and the material laws

(14) $$\begin{array}{cc} J & =\sigma E \end{array}$$

(15) $$\begin{array}{cc} D_{d} & =\varepsilon E, \end{array}$$

the final partial differential equation reads as

(16) ∇·[(σ+jωε)∇V]=0.

In (16), σ is the conductivity tensor and ε is the permittivity tensor. For modeling the electrical conductivity of blood, the so-called eigenvector (EV) model introduced in [11] is used. The EV model computes the inhomogeneous, stress- and shear-rate-dependent, orthotropic conductivity tensor σ as

(17) $$\sigma =\sigma_{\alpha }\left(e_{\alpha }⊗e_{\alpha }\right)+\sigma_{\beta }\left(I-e_{\alpha }⊗e_{\alpha }\right)\;,$$

where $\sigma_{\alpha }$ and $\sigma_{\beta }$ are the principal conductivities depending on the shear rate-dependent degree of alignment of RBCs. The vector $e_{\alpha }$, which characterizes the dominant orientation of RBCs, is obtained from the principal directions, i.e., the eigenvectors of the extra stress tensor. A detailed discussion of the EV method is provided by Jafarinia et al. [11]. The electrical conductivity of the other tissues composing the body segment is modeled as homogeneous, isotropic, and constant in time. In an analogous manner, the permittivity of all tissues is modeled as homogeneous, isotropic, and constant in time.

In the sequel, we employ the complex parameter $\sigma_{c}$, as introduced in [20],

(18) $$\sigma_{c}=\sigma +j\omega \varepsilon ,$$

with which (16) can be written as

(19) $$\nabla \;\cdot \;\sigma_{c}\nabla V=0.$$

### 2.2. Numerical Methods and Simulation

arterioscope.sim generates bioimpedance signals based on patient-specific input parameters. The two main contributors to bioimpedance fluctuations during the cardiac cycle are considered: the blood volume changes in the inspected body segment as well as the time-dependent electrical conductivity of blood. Therefore, arterioscope.sim consists of two modules. Firstly, a CFD module in which the arterial blood flow is simulated, enabling the computation of the electrical conductivity of blood. Secondly, a CEQS module in which the bioimpedance measurement is simulated, including a data interface enabling the interpolation of the blood conductivity tensor field. As the CEQS results do not influence the CFD simulation, the two simulations are performed sequentially.

#### 2.2.1. Computational Fluid Dynamics Simulation

The numerical simulation of the arterial blood flow is implemented in the open-source tool OpenFOAM-5.x [21]. In OpenFOAM, the spatial discretization of (1) and (2) is performed according to the Finite Volume Method (FVM). Hence, the governing equations are integrated over discrete volumes, volume integrals are transformed to surface integrals via the divergence theorem, and those surface integrals are numerically evaluated [22]. The discretized equations are solved by employing the so-called PIMPLE algorithm, which is a combination of the well-known SIMPLE and PISO algorithms used in CFD. It consists of an outer SIMPLE loop and an inner PISO loop, combining PISO’s fast convergence for transient problems with better stability at high Courant–Friedrichs–Lewy (CFL) numbers [23]. The OpenFOAM implementation of the non-Newtonian model introduced in Section 2.1.1 is provided in [17]. The EV model is implemented and combined with the PIMPLE algorithm in OpenFOAM.

The pulsatile blood flow is enforced by assigning a time-dependent velocity in the inlet cross-section of the artery. Assuming a circular cross-section $\Gamma_{\mathrm{inlet}}$, a parabolic velocity profile is prescribed. With the average linear inlet velocity $U_{\mathrm{inlet}}\left(t\right)$ of blood given as a function of time, the velocity perpendicular to the inlet cross-section $u_{⊥}\left(t\right)$ is calculated as

(20) $$u_{⊥}\left(t\right)=2U_{\mathrm{inlet}}\left(t\right)\;\cdot \;\left(1-\frac{r^{2}}{R_{\mathrm{in}}^{2}}\right)\;\;\mathrm{on}\;\;\Gamma_{\mathrm{inlet}},$$

with

(21) $$r^{2}={\left(x-x_{0}\right)}^{2}+{\left(y-y_{0}\right)}^{2}.$$

It is assumed that the velocity at the inlet is purely perpendicular to the cross-section, i.e., the velocity parallel to the inlet cross-section vanishes. The coordinates ($x_{0}$, $y_{0}$, $z_{0}$) denote the center of the inlet cross-section. The inlet velocity $U_{\mathrm{inlet}}\left(t\right)$ was obtained from ultrasound measurements, as discussed in Section 2.2.3.

The CFD simulation is performed for two cardiac cycles as the computed values in the first cycle are still influenced by the initial conditions, which describe no flow. Hence, we only use the conductivity tensor field computed for the second cardiac cycle in the CEQS simulation. Performing the simulation for more cardiac cycles resulted in no significant difference in the conductivity computed in subsequent cycles.

The input parameters for the CFD module are the inlet velocity provided by its temporal Fourier coefficients, parameters of the conductivity model, and geometrical parameters of the artery, as presented in Table 1. The conductivity parameters include the blood density ρ, the hematocrit *H*, the membrane shear modulus μ, and the undeformed RBC aspect ratio $\lambda_{u}$. The geometrical parameters depend on the inspected artery. In the case of the PA, which is the focus of this study, an undeformed artery is modeled as a straight pipe, defined by its length *L* and its radius $\hat{R}$. As the CFD simulation is performed on a static mesh, the radius $\hat{R}$ is chosen to be the artery’s maximum radius during the cardiac cycle, i.e., $\hat{R}=\max\left(R\left(t\right)\right)$.

#### 2.2.2. Computational Electroquasistatics Simulation

The CEQS simulation of the bioimpedance measurement is implemented in the open-source simulation tool openCFS [24]. Based on geometrical and physiological parameters, it generates the bioimpedance signal. The spatial discretization of (19) is conducted according to the Finite Element (FE) method. The boundary conditions are set corresponding to the measurement setup. The electric potential at the reference electrode $\Gamma_{r}$ is set to zero, i.e.,

(22) $$V_{r}=0\;\;\mathrm{on}\;\;\Gamma_{r}.$$

It is prescribed that there is no electric flux between the body surface and the outer domain, i.e.,

(23) $$\sigma_{c}\frac{\partial V}{\partial n}=0\;\;\mathrm{on}\;\;\Gamma_{o},$$

where n is the surface normal vector and $\Gamma_{o}$ is the boundary between the body and outer domain. The injection current $I_{0}$ is prescribed at the injection electrode $\Gamma_{i}$.

**Figure 2:**
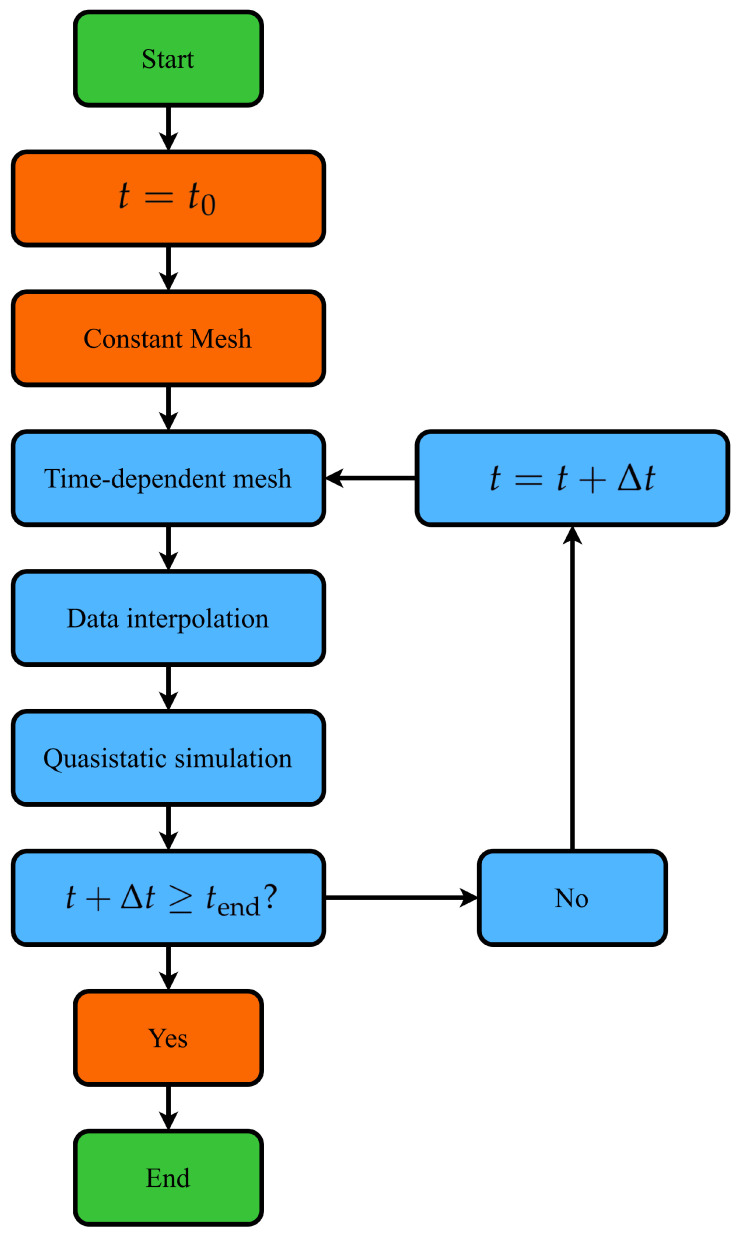
Flowchart of the electrical CEQS simulation. The blue colour represents the simulation loop.

In Figure 2, the flowchart of the CEQS simulation is shown. The mesh consists of two main parts, which are created in Coreform CUBIT 17.1.0 [25]. The first part consists of the artery and the surrounding tissue in close proximity to the artery. This part of the mesh is updated at each time-step to account for the artery’s volume change. The second part consists of the remaining part of the surrounding tissue. This part of the mesh is created at the start of the simulation and is not changed during the computation. Subsequently, the main simulation loop is entered, which is iterated over in each computation time step. First, the time-dependent part of the mesh is created, accounting for the increase and decrease in the artery volume. Then, the computed conductivity tensor field is transferred from the CFD module to the CEQS module, as described in Section 2.2.3. Subsequently, (19) is solved and the transfer impedance is computed as

(24) $$\int_{\Gamma_{\mathrm{in}}}\sigma_{c}\frac{\partial V}{\partial n}\;d\Gamma =I_{0}\;\;\mathrm{on}\;\;\Gamma_{i}.$$

where ${\underline{V}}_{1}$ and ${\underline{V}}_{2}$ are the complex potential values at the pickup electrodes. The input parameters for the CEQS module are related to the material, measurement setup, and geometry, as listed in Table 2. The material parameters include the electrical conductivity and the permittivity of the tissue surrounding the artery. Note that the tissues surrounding the artery, such as bone, skin, and muscle, are modeled as one homogeneous, isotropic material [26]. The measurement parameters include the radius of the current-carrying electrodes and the center-to-center distance between each electrode, as well as the injection current $I_{0}$ and the injection current frequency $f_{J}$. The geometrical parameters depend on the inspected artery. For the PA, the body segment is modeled as a cylinder with a parallel axis to the artery’s axis. The geometry is defined by the radius of the body segment $R_{\mathrm{Ti}}$, its length *L*, and a time series of the artery radius R(t). Additionally, the depth *d* defines the radial distance of the artery’s axis to the electrodes. The electrical conductivity of blood, computed in the CFD module, is also an input of the CEQS simulation.

(25) $$|\underline{Z}|=\frac{|{\underline{V}}_{1}-{\underline{V}}_{2}|}{|{\underline{I}}_{0}|},$$

#### 2.2.3. Data Interpolation

The CFD simulation and the CEQS simulation are conducted on different meshes. The CFD simulation is performed on a fine hexahedral mesh, while the CEQS simulation is performed on a coarser tetrahedral mesh. Hence, the conductivity tensor field resulting from the blood flow simulation has to be interpolated onto the CEQS mesh. At each node *N* of the CEQS mesh, all elements of the conductivity tensor have to be interpolated. This is achieved using the openCFS implementation of Shepard’s method provided in [27]. The conductivity tensor $\sigma_{N}$ at *N* is obtained by a weighted average of the tensors $\sigma_{i}$ at the *K* closest nodes of the CFD mesh, i.e.,

(26) $$\sigma_{N}=\sum_{i=1}^{K}\frac{w_{i}\;\cdot \;\sigma_{i}}{\sum_{i=1}^{K}w_{i}}.$$

The weights $w_{i}$ take into account the distance $r_{i}$ between the target node *N* and the respective source nodes. They are computed as

(27) $$w_{i}={\left(\frac{R_{\max}-r_{i}}{R_{\max}\;\cdot \;r_{i}}\right)}^{m},$$

with $R_{\max}$ being defined as

(28) $$R_{\max}=1.01\;\cdot \;\max\left(r_{i}\right).$$

The weighting can be adjusted with the exponent *m*, considering the limits 1≤m≤3 [27].

The purpose of arterioscope.sim is to generate data of healthy and diseased subjects to study the differences in the bioimpedance signals. Therefore, the first goal is to investigate if simulated CVDs and their pre-existing conditions, arteriosclerosis and atherosclerosis, lead to actual alterations in the computed signals. For this purpose, a patient-specific simulation of a healthy artery acts as a reference. The comparison of the patient-specific simulation with an actual measurement is also meant to be a first validation of arterioscope.sim and the EV model for the electrical conductivity of blood introduced in [11].

### 2.3. Patient-Specific Simulation

In order to compare the patient-specific simulation results with in vivo measurements on corresponding healthy arteries, live IPG measurements on healthy volunteers were performed at the Department of Vascular Diseases at Oslo University Hospital Norway. Ultrasound (US) measurements were performed on the inspected artery of the subject to obtain the required input parameters. The IPG measurements were performed with a Zurich Instruments MFIA device in four-electrode configuration with 4 BNC cables connected to Ambu BlueSensor Q wet-gel electrodes. The transfer impedance was measured at a frequency of 50 kHz, with the excitation voltage being 300 mV. A GE Healthcare Vivid E95 device was used for the US imaging of the arteries, from which the blood velocity and the artery’s geometry were extracted. The measurements were performed on 12 subjects, and the case study with the highest IPG measurements and ultrasound imaging quality was used for this study.

To obtain the artery’s radius time-series from the US video, the radius is measured in multiple cross-sections with the Quipu Cardiovascular Suite, Carotid Studio [28]. The artery radius R(t) is calculated as the average of the radius values at the cross-sections. The average blood velocity in the artery during the US measurement was provided as a plot, from which the values at discrete time steps were extracted. A Fourier series is then fitted to the blood velocity time series to provide the CFD module with the values of the inlet velocity at each computation time step. Along with the IPG and US measurements, patient data, such as the hematocrit *H*, and measurement specifics, such as the radius of the electrodes, the distance between the electrodes, and the injection frequency, are also considered in the simulation. Unknown parameters, including the radius of the body segment $R_{\mathrm{Ti}}$, were assumed. The full parameter lists are given in Table 1 and Table 2.

**Figure 3:**
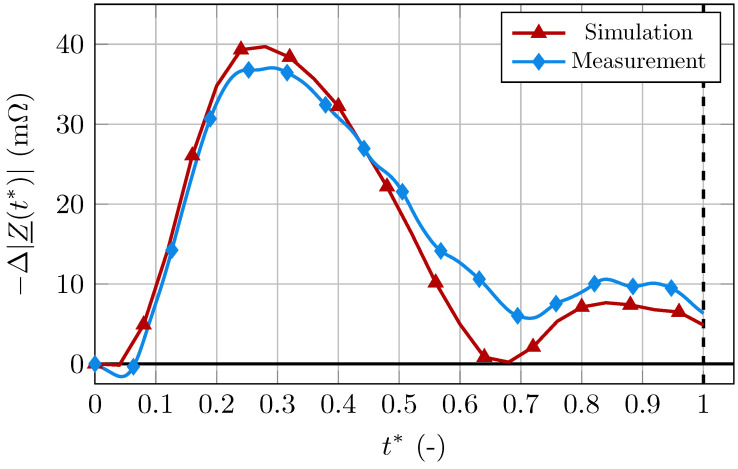
Measured and simulated signal for the PA of one patient.

The simulation results for the PA of one of the subjects are presented and compared to the corresponding measurements in Figure 3. All signals are shown on a normalized time-scale $t^{*}$, defined as

(29) $$t^{*}=\frac{t}{t_{c}},$$

where $t_{c}$ is the length of cardiac cycle.

The base impedance was subtracted, as it is highly dependent on the assumed material and geometry parameters of the surrounding tissue and does not carry information on the physiological changes during the cardiac cycle. It is shown that the simulation matches the measurement reasonably well. In the systolic phase, both signals have their maximum peak at the same time. The magnitude of both peaks is also similar. A second smaller peak during diastole is also visible in both signals, also occurring at the same time. There is a small time-shift between the local minima of the signals between both peaks. Additionally, the minimum of the simulation is approximately 0.008 Ω lower. Possible reasons for those small deviations will be discussed in Section 3.

**Figure 4:**
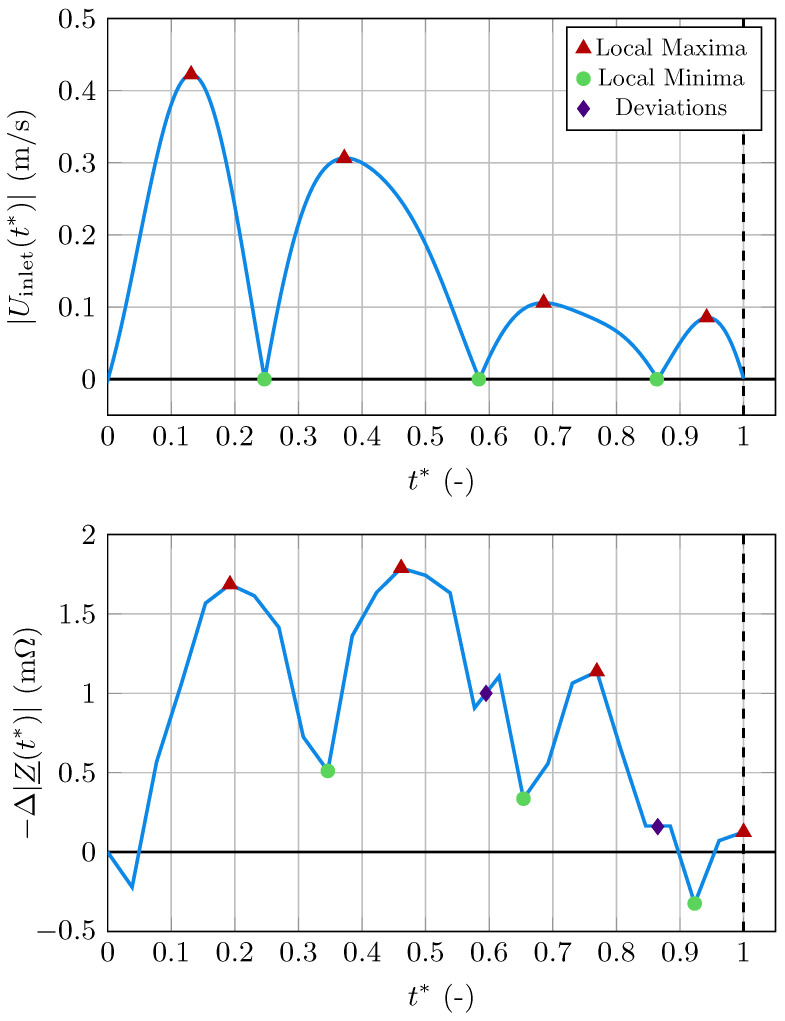
**Top**: Magnitude of the blood velocity. **Bottom**: Bioimpedance changes due to changes in electrical conductivity, i.e., computed IPG signal for fully stiff artery. Local maxima are red circles, local minima are green squares, and points where the signal deviates from the morphology of the velocity magnitude are purple diamonds.

To investigate the influence of the flow-dependent conductivity changes on the signal, we performed a similar simulation with only one adaptation. The radius was set to a constant value representing a fully stiff artery, eliminating all contributions of the blood volume change to the signal. This simulation resulted in a signal representing the bioimpedance changes that stem from the blood conductivity changes. The results for the fully stiff case are presented in Figure 4 along with the magnitude of the blood velocity during the cardiac cycle. Specifically, Figure 4 shows the magnitude of the prescribed average linear velocity in the inlet cross-section $U_{\mathrm{inlet}}\left(t\right)$ according to the velocity recorded during the US measurement. The minima of the velocity magnitude, i.e., the points where the average blood velocity is 0, signify the points in the cardiac cycle at which the sign of the velocity changes. Thus, at these points in time, the blood flow is reversed for a short period of time, which can happen after the systolic peak.

The IPG signal of the fully stiff artery exhibits several local maxima and local minima. The morphology of the signal is similar to the magnitude of the blood velocity within the artery. The reason for this similarity will be discussed in Section 3. However, there is a small time delay between the velocity magnitude and the bioimpedance signal. Additionally, two points where the signal deviates from the morphology of the velocity magnitude are observed. These points are marked as purple diamonds in Figure 4 and their origin will be discussed in Section 3. We quantify the contribution of the changes in electrical conductivity to the healthy bioimpedance signal by comparing the amplitude of the signal in the healthy and fully stiff cases, i.e.,

(30) $$\frac{\left[\max(\Delta \right|\underline{Z}\left(t^{*}\right)\left|\right)-\min(\Delta |\underline{Z}\left(t^{*}\right){\left|)\right]}_{\mathrm{stiff}}}{\left[\max(\Delta \right|\underline{Z}\left(t^{*}\right)\left|\right)-\min(\Delta |\underline{Z}\left(t^{*}\right){\left|)\right]}_{\mathrm{compliant}}}\approx 5\%.$$

Hence, for a healthy artery, the blood volume change is the dominant contributor to the bioimpedance changes.

**Figure 5:**
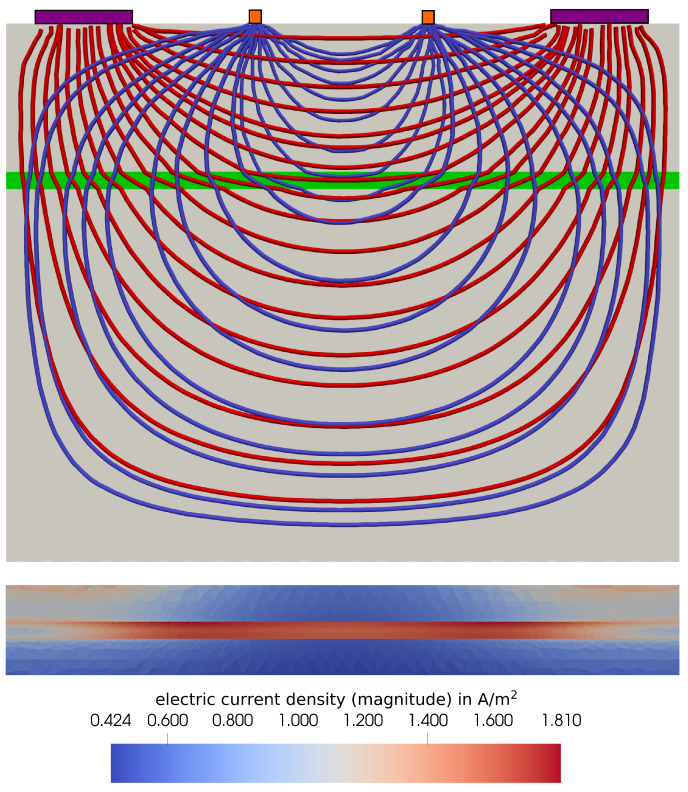
Streamlines of the electric current (red) and the reciprocal current (**blue**) passing through the artery (**green**) and the surrounding tissue (**grey**). **Top**: Cross-section of the whole body segment, including injection electrodes (**purple**) and pickup electrodes/nodes (**orange**). **Bottom**: Close-up of the artery with the magnitude of the electric current density. The images represent the results during systole at $t^{*}=0.16$.

In Figure 5, the streamlines of the electric current and the reciprocal current are visualized. The electric current denotes the current that is actually present in the body during the measurement, when the current is injected at the injection electrodes. The reciprocal current, on the other hand, would be present if the current was injected at the pickup electrodes. The streamlines of the two currents give information on the sensitivity of the measurement to conductivity changes in the artery [29]. This will be further discussed in Section 3. In the current version of arterioscope.sim, the pickup electrodes are replaced by pickup nodes, i.e., the surface of the pickup electrodes is not modeled. Future updates on the platform will include modeling of the pickup surfaces. Additionally, the magnitude of the electric current density in and around the artery is visualized in Figure 5.

**Figure 6:**
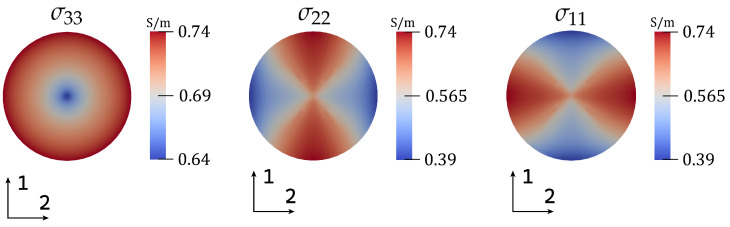
Main diagonal elements of electrical conductivity tensor in a cross-section perpendicular to the flow direction (3-direction) during systole at $t^{*}=0.16$.

The electrical conductivity tensor field inside of the artery is visualized in Figure 6. The main diagonal elements of the tensor are shown in a cross-section perpendicular to the flow direction at $t^{*}=0.16$, i.e., during the systolic phase. While the results for the main diagonal element corresponding to the flow direction (3-direction) are rotationally symmetric, the other main diagonal elements are not due to the anisotropic behavior of blood’s electrical conductivity. The largest electrical conductivity in flow direction is observed at the artery wall and nearby it, while the lowest values are found close to the artery’s longitudinal axis.

### 2.4. Arteriosclerosis

We introduce arteriosclerosis, i.e., increased stiffness of the arterial wall, into the model by adjusting the amplitude of the artery radius R(t). All other parameter values are equal to the healthy reference simulation presented in Section 2.3. The generated signals are then compared with the healthy reference signal to detect alterations in the signals depending on the stiffness level. The radius $R_{X}\left(t^{*}\right)$ for a diseased artery with decreased compliance is defined as

(31) $$R_{X}\left(t^{*}\right)=R\left(t^{*}=0\right)+\frac{X}{100}\;\cdot \;\left(R\left(t^{*}\right)-R\left(t^{*}=0\right)\right),$$

where $R(t^{*})$ is the radius curve. With (31), we specify the radius amplitude of an artery with $C_{X}\%$ compliance to be X% of a healthy radius amplitude. Hence, X=100 for a healthy artery and X=0 for a completely stiff artery. The relative compliance $C_{x}$ is defined as the ratio of the actual volume change and the healthy volume change, i.e.,

(32) $$C_{X}=\frac{R_{X}{\left(t^{*}\right)}^{2}}{R{\left(t^{*}\right)}^{2}}.$$

**Figure 7:**
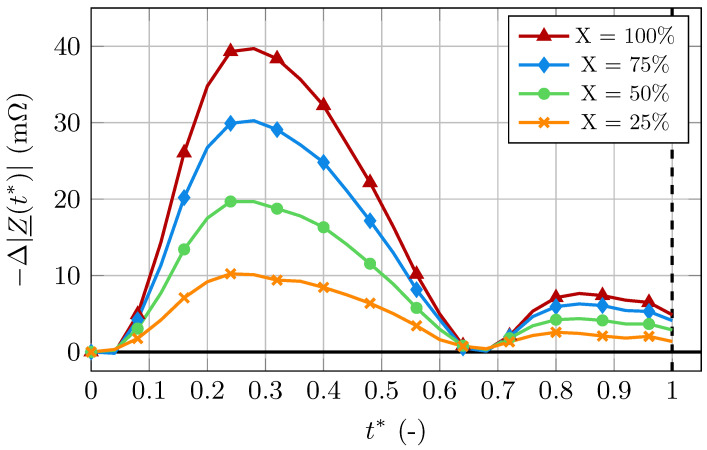
Generated signals for different degrees of stiffness.

The generated bioimpedance curves for four different compliance levels are presented in Figure 7, clearly showing that the signal’s amplitude decreases with the stiffness level. Note that the simulated bioimpedance curves for X=0, i.e., a fully stiff artery, are shown in Figure 4. We quantify this decrease with the quantity $\beta_{X}$, defined as

(33) $$\beta_{X}=\left(1-\frac{\left[\max(-\Delta \right|\underline{Z}\left(t^{*}\right){\left|)\right]}_{X}}{\left[\max(-\Delta \right|\underline{Z}\left(t^{*}\right){\left|)\right]}_{100}}\right)\;\cdot \;100\;\;\left[\%\right].$$

The values of $\beta_{X}$ for the chosen levels of compliance are listed in Table 3.

**Figure 8:**
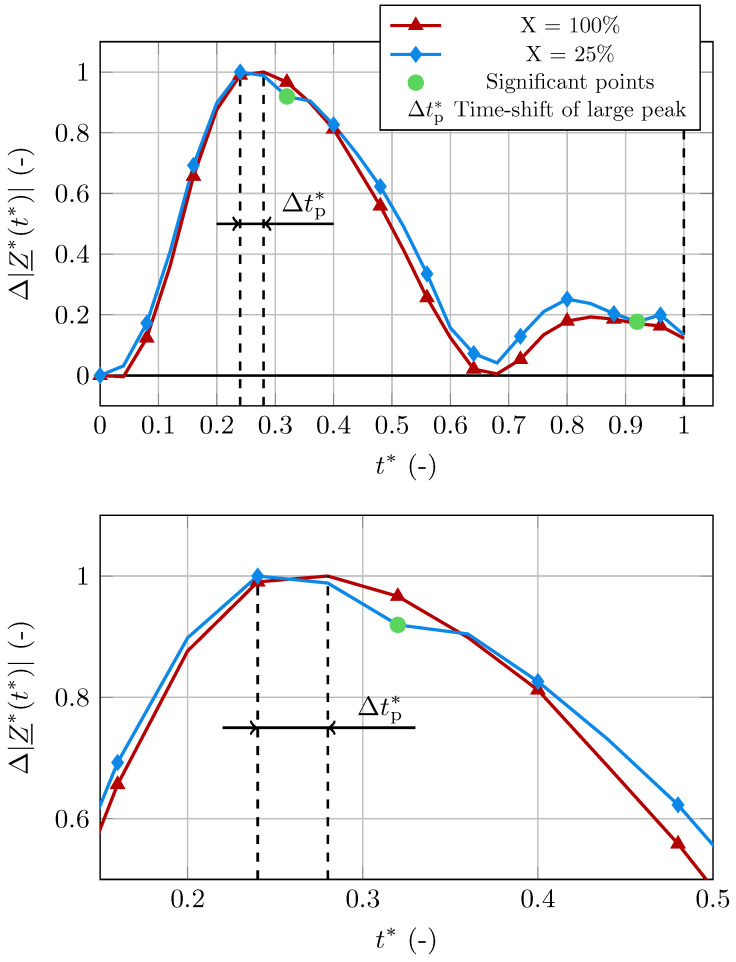
Comparison of the waveform for a healthy subject and a subject with increased wall stiffness. **Top**: Additional significant points (**green circles**) are observed in the signal with increased stiffness. **Bottom**: The large peak of the signal with healthy compliance happens later in the cardiac cycle.

In addition to the changes in the signal’s amplitude, we observed alterations in its waveform. These changes are presented in Figure 8, where the healthy curve (X=100) and a curve with increased stiffness (X=25) were normalized with respect to their peak value. The normalized, non-dimensional impedance is defined as

(34) $$\Delta |{\underline{Z}}^{*}\left(t^{*}\right)|=\frac{-\Delta |{\underline{Z}}^{*}\left(t^{*}\right)|}{\max(-\Delta |{\underline{Z}}^{*}\left(t^{*}\right)|)}.$$

**Figure 9:**
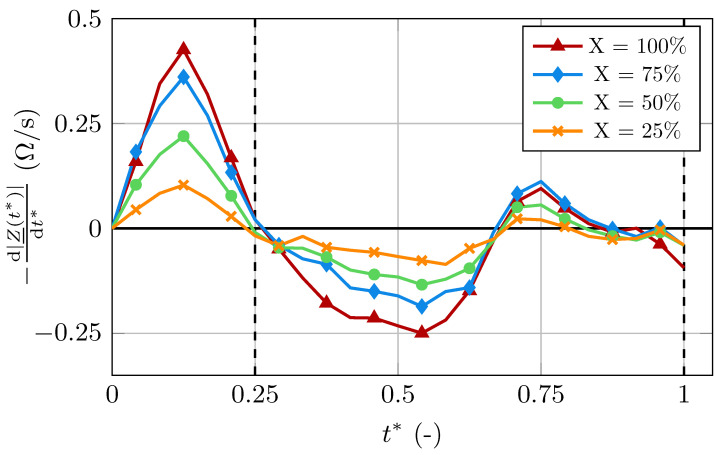
Temporal derivative of the signal in the healthy case and with increased wall stiffness.

The signal for the less compliant arterial wall shows additional significant points that are not visible in the healthy signal. Specifically, these points are observed to happen directly after the systolic peak and after the second peak, where a local minimum arises, which does not appear in the healthy signal. Additionally, the magnitude of the small peak relative to the large peak is greater in the stiff case. We also observe the large peak happening earlier in the cardiac cycle for increased stiffness. Specifically, in the less compliant case, the large peak happens at $t^{*}=0.24$, while it appears at $t^{*}=0.28$ in the healthy case. This shift of the systolic peak can also be deduced from the temporal derivative of the signal, as shown in Figure 9. Evaluating the temporal derivative at $t^{*}=0.25$ gives

(35) $${\left[-\frac{d|\underline{Z}\left(t^{*}\right)|}{dt^{*}}\right]}_{X=100}>{\left[-\frac{d|\underline{Z}\left(t^{*}\right)|}{dt^{*}}\right]}_{X=75}>0$$

and

(36) $$0>{\left[-\frac{d|\underline{Z}\left(t^{*}\right)|}{dt^{*}}\right]}_{X=50}>{\left[-\frac{d|\underline{Z}\left(t^{*}\right)|}{dt^{*}}\right]}_{X=25},$$

as the peak happens before $t^{*}=0.25$ for X=25,50 and after $t^{*}=0.25$ for X=75,100.

### 2.5. Atherosclerosis

**Figure 10:**
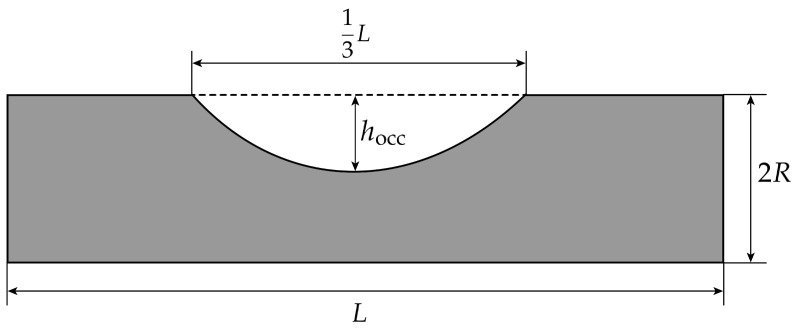
Geometrical parameters of the artery with a stenosis.

To introduce atherosclerosis—i.e., deformations of the artery caused by plaque formation at the arterial wall—into the model, the geometry parameters of the platform are extended by the parameter $h_{\mathrm{occ}}$. It describes the maximum depth of the arterial stenosis, as seen in Figure 10.

Hence, an artery with Y% occlusion is defined by

(37) $$h_{\mathrm{occ}}=\frac{Y}{100}\;\cdot \;2R.$$

**Figure 11:**
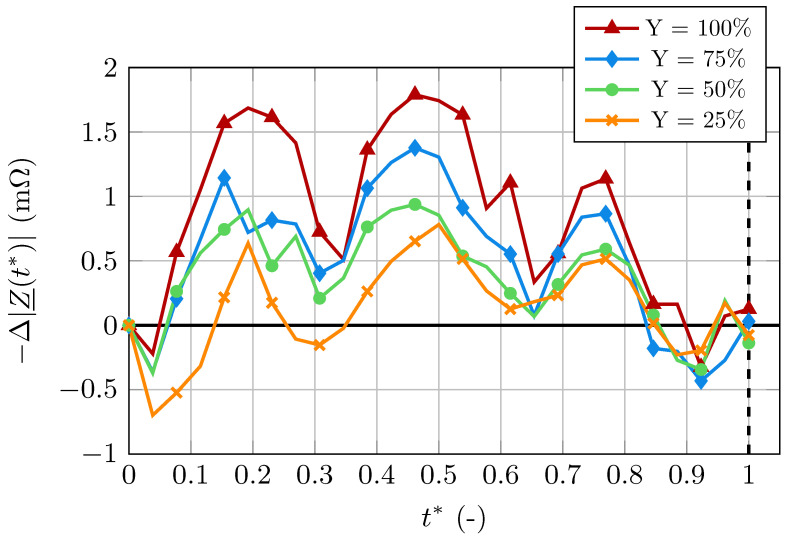
Computed IPG signals for different levels of occlusion.

The computed IPG signals for four different levels of occlusion are shown in Figure 11. The artery deformation influences the blood flow and, therefore, the electrical conductivity of blood. To visualize its effect on the bioimpedance without it being overshadowed by the dominant blood volume change, the simulations were performed on a completely stiff artery with a constant radius.

We observe that the signal changes depending on the level of occlusion, as all four signals show significant differences. Firstly, the magnitude of the signal’s peaks decreases with increasing size of the stenosis. Secondly, the local minimum between the first and second peak gets lower with increasing occlusion. In addition to the changes in amplitude, we can observe changes in the waveform of the signal. The first and second peak have a broad, smooth shape for the artery with no stenosis. These peaks become increasingly sharp for high levels of occlusion.

## 3. Discussion

### 3.1. Patient-Specific Simulation

As seen in Figure 3, the simulated and measured signals match very well. With this comparison, we show that arterioscope.sim is able to produce an IPG signal with a similar morphology to an actually measured signal, based on patient-specific input parameters. An exact match would be unrealistic due to the simplifications and uncertainties in the model. Additionally, the main purpose of the simulation platform is not to obtain an exact match between simulation and measurement but, rather, to investigate possible alterations in the signal due to different pathologies. For this purpose, we deem the simulation results to be sufficiently close to the measurements on a healthy patient.

The small differences that are visible in Figure 3, especially at the local minima of the signals, could be due to geometrical simplifications of the artery and the corresponding averaging of the measured radius values. However, modeling inaccuracies such as neglecting smaller contributors to the bioimpedance changes, e.g., time-dependent and inhomogeneous properties of the surrounding tissues, can be a further cause to the signal differences. This also includes anisotropic properties of the surrounding tissues, which are not included in the simulation. Anisotropic and inhomogeneous electrical conductivity of the tissue that is not considered in the simulation could cause different current paths in the actual tissue than in the simulation. Additionally, the compression of the surrounding tissue can lead to time-dependent changes in the properties of surrounding tissues, which is neglected in the model.

The simulation of the fully stiff artery resulted in a signal with morphology similar to the blood velocity magnitude in the artery, as seen in Figure 4. Velocities with high magnitude lead to large velocity gradients adjacent to the artery wall. The resulting shear stresses align the RBCs approximately with the flow direction. However, this alignment is independent of the sign of the velocity. Therefore, we compare the morphology of the velocity magnitude with the signal. Aligned RBCs cause high electrical conductivity in the flow direction. We conclude this conductivity to be the dominant element of the conductivity tensor as the electric field is mostly parallel to the flow direction. Additionally, Figure 5 shows that the electric current in the artery is mostly parallel to the flow direction in the segment between the artery cross-sections in the plane perpendicular to the body surface at the pickup electrodes. Hence, the bioimpedance signal closely follows the velocity magnitude curve. The time delay between velocity magnitude and the signal could result from a variety of causes. Firstly, when the velocity decreases in pulsatile flow, the velocity profile deviates from the parabolic profile and flow reversals appear. This causes delayed disorientation of RBCs in the deceleration phase of the blood flow. phase [30]. Secondly, while the conductivity in the flow direction is the dominant element of the tensor, other elements also matter for the electric current, especially when entering and leaving the artery. The increase in velocity magnitude leads to a decrease in the conductivity in a specified direction perpendicular to the flow direction, as the RBCs align their largest surface perpendicular to this direction. Therefore, the bioimpedance changes result from a trade-off between increases and decreases in the elements of the conductivity tensor, which can also lead to a time delay between the velocity magnitude and bioimpedance signal. The points in the signal where it deviates from the velocity magnitude’s morphology could either also result from this trade-off or from the non-linear dependency between velocity and conductivity. These non-linearities are caused by the aforementioned deviation of the velocity from a purely parabolic flow profile [30]. Note that the time resolution in the CFD module is significantly higher than in the CEQS module to ensure stability in the CFD simulation. However, increasing the time resolution in the CEQS module is planned for future extensions of arterioscope.sim to obtain smoother signals from the simulations.

In Figure 5, the electric current and reciprocal current streamlines are visualized. As introduced, the reciprocal current appears when injecting the current at the pickup electrodes [29]. We make several observations from the plot. Firstly, we observe that the electric current shows discontinuities at the artery wall. This underlines the importance of the detailed modeling of the anisotropic nature of the electrical conductivity of blood. The electrical conductivity in the flow direction is higher than the conductivity perpendicular to the artery wall due to the alignment of RBCs with the flow. Hence, when the electric current enters the artery, it gets redirected closer to the flow direction due to the high conductivity. Wherever the current leaves the artery, the opposite effect is observed. Modeling the electrical conductivity of blood as isotropic would neglect this effect completely. Secondly, the angle between the electric current and reciprocal current is a measure of the local sensitivity. If the streamlines are perpendicular to each other, the measured impedance is insensitive to local changes of the conductivity. The smaller the angle between the streamlines is, the higher the sensitivity of the measured impedance to the local changes of conductivity [29]. In the simulation, the artery is the only part of the domain in which the conductivity changes. Hence, the sensitivity in the artery is especially important. We can observe the highest sensitivity in the artery between the pickup electrodes. Here, the electric current and reciprocal current are almost parallel.

The electric current density in the artery and around it is also visualized in Figure 5. Firstly, we observe that the magnitude is significantly higher in the artery than in the surrounding tissue. This is due to the high electrical conductivity of blood in comparison to the surrounding tissues. Secondly, we observe that, at an arbitrary cross-section of the artery, the magnitude of the electric current is higher in areas close to the artery wall. This can be attributed to the alignment of RBCs close to the wall, as the large velocity gradients constitute high shear rates leading to the alignment and increase in conductivity in the flow direction, which we observe in Figure 6.

### 3.2. Arteriosclerosis

The computed IPG signals for different levels of stiffness showed significant differences. With increasing stiffness, the artery experiences smaller volume changes. Because the blood volume changes are the dominant contributor to the bioimpedance changes, the amplitude of the IPG signal decreases significantly with increasing stiffness. The arterial volume is proportional to the square of the radius. However, when decreasing the maximum radius during the cardiac cycle, the signal’s amplitude decreases almost linearly, as seen in Table 3. Firstly, this could be due to the small radius fluctuations during the cardiac cycle, where the quadratic dependency is close to a linear one. Secondly, the signal is not solely dependent on the volume change but also on the changes in electrical conductivity. While the decrease in amplitude is the most obvious result of increases in stiffness, its interpretation proves to be difficult. This is because the amplitude is also dependent on a number of other factors, such as the exact geometry and composition of the body segment and the material parameters of the surrounding tissue, which are mostly unknown.

Therefore, the observed alterations in the waveform could be an additional important indicator for increased stiffness. As mentioned before, the bioimpedance changes stem from two sources: the changes in blood volume and the changes in electrical conductivity. As the stiffness increases, the influence of the volume changes on the signal decreases while the influence of the conductivity changes increases. This explains the alterations in the waveform. In the signal with less compliance, new significant points were observed. These dents happen at points in time when the local minima in the completely stiff case appear due to the changes in the electrical conductivity, as seen in Figure 4. These local minima are overshadowed by the bioimpedance changes caused by the volume change in the healthy case, but appear in the IPG signal as the stiffness increases, due to the increased influence of the electrical conductivity of blood on the signal.

The increased influence of the electrical conductivity on the IPG signal with increased stiffness also explains the time-shift of the signal’s large peak. When only assessing the bioimpedance changes due to electrical conductivity changes, i.e., in the completely stiff case, the large peak happens earlier in the cardiac cycle than in the healthy case. This is due to the blood volume peak happening later in the cycle. As the influence of the electrical conductivity changes increases with increased stiffness, the large peak of the signal moves to earlier points in the cardiac cycle, which can be observed in Figure 7 and Figure 9.

We conclude that introducing arteriosclerosis into the simulation platform leads to alterations in the signal’s amplitude and waveform. These changes are dependent on the level of introduced stiffness. The changes of the waveform may be underestimated in the shown simulations. This is because the same inlet velocity was prescribed for different levels of stiffness. However, increased stiffness could lead to large velocity peaks, because stiff arterial walls dampen the flow less. Therefore, larger velocity magnitudes could result in larger differences in the signal waveforms.

### 3.3. Atherosclerosis

The simulations presented for atherosclerosis were performed on fully stiff arteries, neglecting the unrelated contributions of the volume change. However, arteriosclerosis commonly precedes atherosclerosis, i.e., high levels of stiffness commonly coexist with atherosclerotic deformations [2]. Still, the presented bioimpedance curves would be superpositioned with the curves resulting from the blood volume change in a measured signal.

Most importantly, we can conclude that the introduction of atherosclerosis into the simulation leads to alterations in the computed signals. These alterations become more pronounced as the level of arterial occlusion increases. However, due to the complex relationships between velocity, flow direction, electrical conductivity, and bioimpedance, computer-aided in-depth analysis will give insights into the exact causes of changes resulting from the stenosis. At this point, a large number of causes for these changes is possible, such as the decrease in blood volume due to the stenosis, the change in flow direction, and the high-velocity magnitudes in the small cross-section in the occluded area.

## 4. Conclusions and Future Work

In this study, we introduced the simulation platform arterioscope.sim—consisting of a CFD module and a CEQS module—to simulate bioimpedance measurements using patient-specific input parameters. The blood flow simulation in the CFD module enables the computation of the time-dependent and anisotropic electrical conductivity of blood, which is needed in the CEQS simulation of the bioimpedance measurement. The goal is to investigate if the introduction of atherosclerosis and arteriosclerosis would lead to alterations in the computed signals. To generate a healthy reference signal, US imaging of a patient’s artery was performed to extract patient-specific input parameters. The comparison of the generated and measured signals from the same patient showed that arterioscope.sim can produce a bioimpedance signal with similar morphology to a measured signal if provided with patient-specific parameters. Introducing atherosclerosis and arteriosclerosis into the simulations leads to visible changes in the generated signals, depending on the progression of either disease. This is an essential observation, as the platform is to be used to create bioimpedance signals of virtual patients, either in a healthy state or with a disease, to allow for studies of these alterations in the signals.

This study shall not act as a complete validation of arterioscope.sim—rather, the goal of this study is to confirm that simulated diseases of arteries result in significant alterations in the simulated signals. This confirmation justifies efforts to statistically validate the model. As this goal was reached, future work on arterioscope.sim will include the validation of the framework by comparing patient-specific simulations and measurements on a larger number of subjects. Additionally, after generating many signals, the possibility of replacing parts of the platform with surrogate models will be investigated. The application of classification algorithms in diagnosing arterial diseases, trained and tested on signals generated by the platform, will also be a priority.

Future investigations will include the influence of pulsatile volumes surrounding the artery. Additionally, the frequency dependency of the measurement and its sensitivity concerning blood volume and conductivity changes will be investigated. The influence of electrode positioning and orientation will also be examined.

## Figures and Tables

**Table 1:** Parameters for blood flow simulation.

Parameter	Symbol	Value	Unit
Blood density	ρ	1060	kg/m^3^
Hematocrit	*H*	0.39	[-]
Membrane shear modulus	μ	$10^{-5}$	kg/s^2^
Short RBC semiaxis	*a*	$1.52\times 10^{-6}$	m
Long RBC semiaxis	*b*	$4\times 10^{-6}$	m
Undeformed RBC aspect ratio	λ	0.38	[-]
Length	*L*	0.15	m
Artery radius (CFD)	$\hat{R}$	0.0022025	m

**Table 2:** Parameters for bioimpedance simulation.

Parameter	Symbol	Value	Unit
Depth	*d*	0.03	m
Length	*L*	0.15	m
Radius of surrounding tissue	$R_{\mathrm{Ti}}$	0.06	m
Electrode distance	$l_{\mathrm{el}}$	0.038	m
Electrode radius	$R_{\mathrm{el}}$	0.0095	m
Injection current density	$J_{0}$	15	A/m^2^
Injection current frequency	$f_{J}$	50	kHz
Electrical conductivity of surrounding tissue	$\sigma_{\mathrm{Ti}}$	0.23	S/m
Permittivity of surrounding tissue	$\varepsilon_{\mathrm{Ti}}$	$5.31\times 10^{-8}$	As/Vm
Permittivity of blood	$\varepsilon_{\mathrm{bl}}$	$4.6\times 10^{-8}$	As/Vm

**Table 3:** Decrease in amplitude for different levels of compliance.

X% Compliance	$\beta_{X}\;\%$
100 (healthy)	0
75	23.714
50	50.374
25	74.219

## Data Availability

Data are contained within the article.

## Abbreviations

The following abbreviations are used in this manuscript:

IPG	Impedance plethysmography
RBC	Red blood cell
CFD	Finite Element
CEQS	Computational Electroquasistatics
FVM	Finite Volume Method
EV	Eigenvector
